# Effect of Antimicrobial Divalent Metal Cations Onto Oxidized Surface of Polyhydroxyalkanoate Films on Biodegradability in Seawater

**DOI:** 10.1002/mabi.202500162

**Published:** 2025-08-26

**Authors:** Jobu Tateiwa, Yu‐I. Hsu, Hiroshi Uyama, Takeharu Tsuge, Tadahisa Iwata

**Affiliations:** ^1^ Department of Biomaterials Sciences Graduate School of Agricultural and Life Sciences The University of Tokyo Bunkyo‐ku Tokyo Japan; ^2^ Department of Applied Chemistry Graduate School of Engineering Osaka University Suita Osaka Japan; ^3^ Department of Materials Science and Engineering Institute of Science Tokyo Yokohama Kanagawa Japan

**Keywords:** antimicrobial metal ions, biodegradable plastic, biodegradation control, polyhydroxyalkanoates, surface modification

## Abstract

Poly[(*R*)‐3‐hydroxybutyrare‐*co*‐(*R*)‐3‐hydroxypivalate] (P(3HB‐*co*‐3HPi)) films, a type of polyhydroxyalkanoate (PHA), are oxidized using photoactivated chlorine dioxide radical (ClO_2_•) gas to generate carboxyl groups and loaded with divalent metal cations, including Cu^2+^, Zn^2+^, and Ca^2+^ ions, via ionic interactions. The P(3HB‐*co*‐3HPi) films loaded with Cu^2+^ ions exhibit enhanced antibacterial activity against Gram‐positive bacteria (*Staphylococcus aureus*) and Gram‐negative bacteria (*Escherichia coli*) compared with untreated P(3HB‐*co*‐3HPi) films. In seawater, the biodegradation of these Cu^2+^ and Zn^2+^‐loaded films is initially inhibited by the antimicrobial activity of the cations and occurs gradually; therefore, loading antimicrobial divalent metal cations onto the surface of PHAs inhibits biodegradation in seawater temporarily but allows biodegradation to occur with time. These results indicate that PHAs could be employed in seawater without undergoing biodegradation, such that PHAs could be used in fishing gear, including fishing lines that are repeatedly exposed to seawater for short periods.

## Graphical abstract

Divalent metal cations such as copper (II) ions are loaded on the surface of poly[(*R*)‐3‐hydroxy‐*co*‐(*R*)‐3‐hydroxypivalate] films that are oxidized by photoactivated chlorine dioxide radical gas. These films exhibited enhanced antibacterial activity against Gram‐positive and Gram‐negative bacteria. In seawater, the films maintained their antimicrobial activity at the initial stage, but the biodegradation of the films occurred with the passage of time.

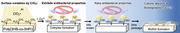

## 1 Introduction

In recent years, plastic waste has become a serious marine pollutant, and developing biodegradable plastics that microorganisms can degrade has become a research focus [1]. Polyhydroxyalkanoates (PHAs) are particularly relevant biodegradable plastics in these applications because they biodegrade under various environmental conditions, including soils, lakes, and oceans [2].

PHAs are biosynthesized by microorganisms from sugars and vegetable oils [3]. A plethora of studies on developing PHAs, such as poly[(*R*)‐3‐hydroxybutyrate] and its copolymer poly[(*R*)‐3‐hydroxybutyrate‐*co*‐(*R*)‐3‐hydroxyhexanate] (PHBH) have been conducted [4, 5]. For example, Iwata et al. developed PHAs fibers with a high tensile strength exceeding 1 GPa via stretching at room temperature after ice bath stretching and annealing the fiber [6]. Omura et al. developed elastic PHA fibers by melt spinning, leaving a small amount of crystals unmelted [7].

Despite the development of these high‐performance PHA materials, their excellent biodegradability renders their continuous application under environmental conditions difficult. For example, since fishing gear is a major source of plastic waste, expanding the potential application of PHAs into fishing gear is highly desirable; however, it is necessary to control biodegradation so that these materials are not biodegraded when in use.

In general, the biodegradation of PHA materials is initiated by microorganisms on the material surface, and surface modification can be effective in controlling biodegradation [8, 9]. In our recent study, the surface of PHBH films was oxidized using photoactivated chlorine dioxide radical (ClO_2_•) gas to generate carboxyl groups, and crystal violet (CV), an antimicrobial quaternary ammonium cation, was loaded via ionic interactions [10]. The antimicrobial properties of CV successfully inhibited the biodegradation of PHBH in soil. In addition, this surface modification did not significantly affect the physical properties of the material [11]. In contrast, CV has minimal antimicrobial activity against Gram‐negative bacteria, so biodegradation was not inhibited in aqueous environments where Gram‐negative PHA‐degrading bacteria are dominant [10, 12, 13].

Herein, antimicrobial metal cations were loaded onto the surface of oxidized PHA films to temporarily inhibit PHA biodegradation in aqueous environments such as seawater. Divalent metal cations such as Cu^2+^ and Zn^2+^ are commonly used because they are inexpensive and exhibit low toxicity to the human body [14]. The mechanisms underlying these cations' antimicrobial activity have been investigated in various studies but remain unclear [15]. However, several important factors related to their antimicrobial activity have been identified. For example, Cu^2+^ ions generate reactive oxygen species (ROS) upon contacting bacteria that irreversibly damage bacterial membranes [16, 17]. Notably, these cations exhibit antimicrobial activity against both Gram‐positive and Gram‐negative bacteria [18].

**FIGURE 1:**
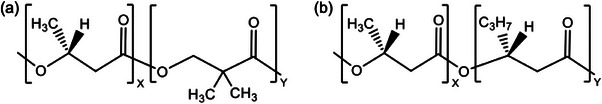
Molecular structures of the PHAs used in this study: (a) poly[(*R*)‐3‐hydroxybutrate‐co‐(*R*)‐3‐hydroxypivalate] (P(3HB‐*co*‐3HPi)) and (b) poly[(*R*)‐3‐hydroxybutyrate‐*co*‐(*R*)‐3‐hydroxyhexanate] (PHBH).

In this study, antimicrobial divalent metal cations were loaded onto the surface of PHAs through ionic interactions with carboxyl groups generated by surface oxidation with photoactivated ClO_2_• gas. Figure 1 shows the molecular structures of the PHA molecules used in this study. Whether the loading of these cations enhanced the antimicrobial activity of the films against Gram‐positive and Gram‐negative bacteria was investigated. In addition, the effect of loading divalent metal cations on the biodegradability of the films was investigated in seawater.

## 2 Results and Discussion

### 2.1 Loading of Divalent Metal Cations

**FIGURE 2:**
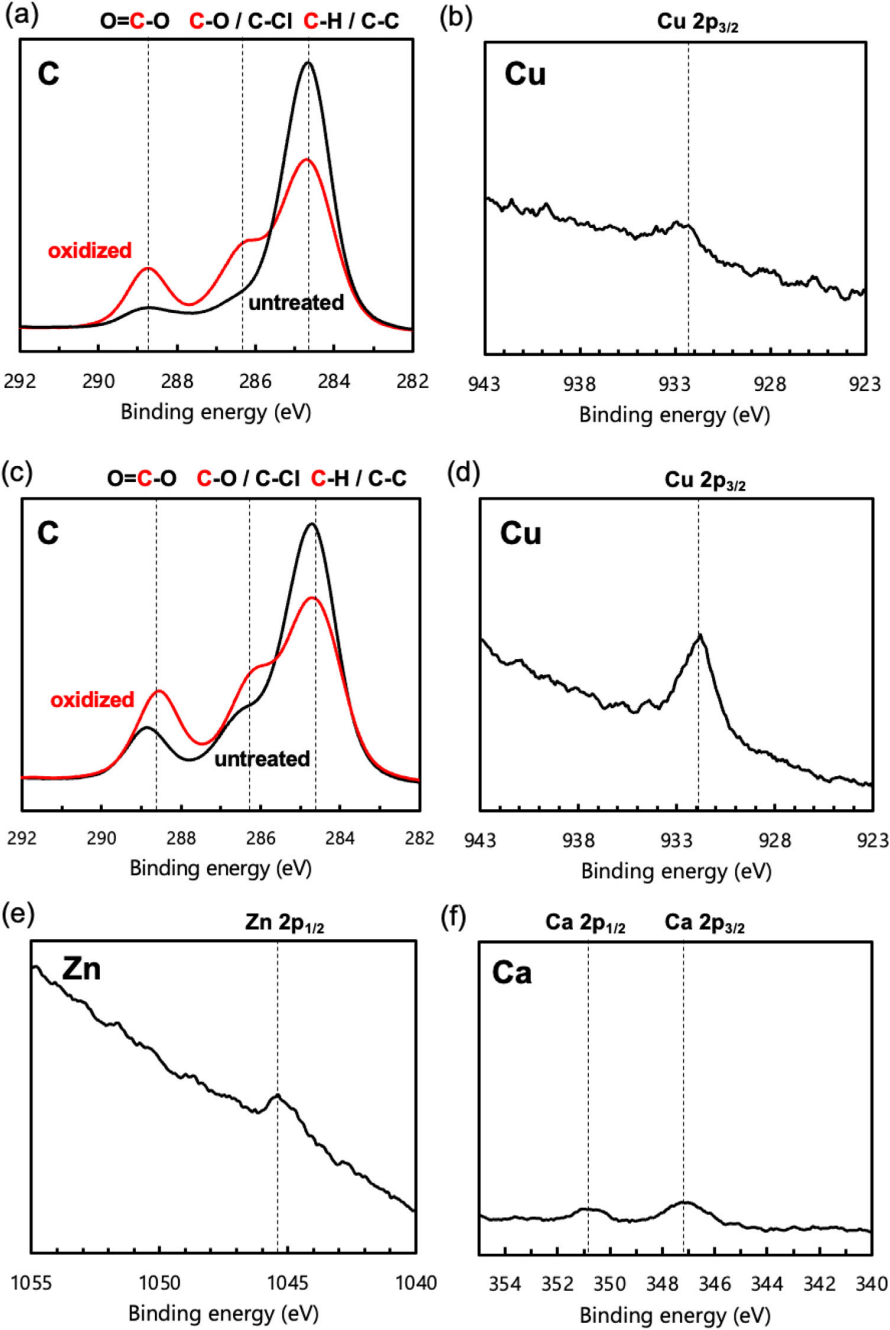
XPS spectra: (a) PHBH films before and after oxidation via photoactivated ClO_2_• gas, (b) oxidized PHBH films immersed in copper (II) bromide aqueous solution, (c) P(3HB‐co‐3HPi) films before and after oxidation bia photoactivated ClO2• gas, (d) oxidized P(3HB‐co‐3HPi) films immersed in copper (II) bromide aqueous solution, (e) oxidized P(3HB‐co‐3HPi) films immersed in zinc (II) bromide aqueous solution, and (f) oxidized P(3HB‐co‐3HPi) films immersed in calcium chloride aqueous solution.

The surface of PHBH films before and after oxidation via photoactivated ClO_2_• gas was analyzed using X‐ray photoelectron spectroscopy (XPS). Similar to previous studies, the peak intensities derived from C─C/C─H (284.7 eV) decreased after oxidation, while the peak intensities derived from C─O/C─Cl (286.5 eV) and O─C═O (289.0 eV) increased, confirming carboxyl group formation (Figure 2a) [10, 11, 19, 20]. Then, loading Cu^2+^ ions onto the oxidized films was attempted. The XPS spectra for Cu atoms on the surface showed a slight increase in a peak derived from Cu 2p_3/2_ (932 eV); however, the intensity was near the baseline, indicating that Cu^2+^ ions were not loaded across the entire surface of the oxidized PHBH film (Figure 2b). This result was unexpected because an increase in the peak associated with Cu atoms was detected using XPS in the case of TEMPO‐oxidized cellulose that also had carboxyl groups on the material surface [21].

The minimal Cu^2+^ ions loading onto PHBH was hypothesized to result from the PHBH surface that lacks adequate sites for stable Cu^2^⁺ coordination. Therefore, replacing PHBH with another PHA could be used to address this situation.

**FIGURE 3:**
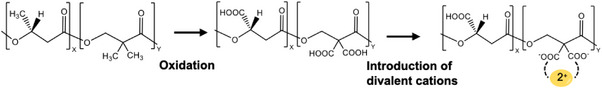
Schematic illustration of divalent cations loading onto oxidized P(3HB‐*co*‐3HPi) in the form of a complex.

If two adjacent carboxyl groups were present on the PHA film, divalent cations could be loaded in the form of complexes; therefore, PHBH was replaced with poly[(*R*)‐3‐hydroxy‐*co*‐(*R*)‐3‐hydroxypivalate] (P(3HB‐*co*‐3HPi)), containing two adjacent methyl groups. These methyl groups were converted to carboxyl groups through oxidation using photoactivated ClO_2_• gas and loaded with Cu^2+^ ions via complex formation (Figure 3).

The analysis of the surface of P(3HB‐*co*‐3HPi) films before and after oxidation using photoactivated ClO_2_• gas via XPS confirmed the formation of carboxyl groups similar to PHBH (Figure 2c). The decrease in the molecular weight of P(3HB‐*co*‐3HPi) powders and films after processing or oxidation was not confirmed (Figure S1), indicating that processing and surface oxidation do not affect the stability of P(3HB‐*co*‐3HPi) molecules. Then, the loading of Cu^2+^ ions was then attempted. The XPS spectra for Cu atoms on the surface showed a clear peak derived from Cu 2p_3/2_, dissimilar to Cu^2+^‐loaded PHBH (Figure 2d); therefore, the replacement of PHBH with P(3HB‐*co*‐3HPi) allowed Cu^2+^ ions to be loaded across the entire surface of the oxidized film. Furthermore, Zn^2+^ and Ca^2+^ ion loading was also attempted. The XPS spectra for Zn and Ca atoms showed peaks derived from Zn 2p_1/2_ (1045 eV) and Ca 2p_1/2_ (351 eV) and Ca 2p_3/2_ (347 eV), respectively; therefore, these cations were also loaded onto the P(3HB‐*co*‐3HPi) films (Figure 2e,f).

### 2.2 Antibacterial Tests

Table 1 shows the results of the antimicrobial tests on the P(3HB‐*co*‐3HPi) film and P(3HB‐*co*‐3HPi)‐Cu film. After exposure to the tested bacteria, P(3HB‐*co*‐3HPi)‐Cu films showed a significant reduction in viable bacterial counts after 24 h compared with the untreated P(3HB‐*co*‐3HPi) films. Based on these bacterial counts, the antimicrobial activity value (*R*) was calculated. *R* is an index for evaluating antimicrobial efficacy in JIS Z 2801 [22]. The R value was calculated according to the following formula.

$$R=U_{t}-A_{t}$$

where *U_t_* is the logarithmic mean of the number of viable bacteria after 24 h on the P(3HB‐*co*‐3HPi) film, and *A_t_* is the logarithmic mean of the number of viable bacteria after 24 h on the P(3HB‐*co*‐3HPi)‐Cu films. An antimicrobial effect is recognized if the *R* value exceeds 2, i.e., the bacterial growth rate is less than 1/100 of the control.

**TABLE 1:** Bacterial counts and antimicrobial efficacies of P(3HB‐*co*‐3HPi) and P(3HB‐*co*‐3HPi)‐Cu films in antimicrobial tests against *Staphylococcus aureus* and *Escherichia coli*.

Tested bacteria	Sample	Bacterial count (cfu/cm^2^)			Logarithm of the mean of bacterial count	Antimicrobial efficacy *R*
		*n* = 1	*n* = 2	*n* = 3		
*Staphylococcus aureus*	P(3HB‐*co*‐3HPi)	3.9 × 10^2^	5.1 × 10^2^	5.4 × 10^2^	2.7	–
	P(3HB‐*co*‐3HPi)‐Cu	<2.5	<2.5	<2.5	0.4	2.3
*Escherichia coli*	P(3HB‐*co*‐3HPi)	3.8 × 10^5^	4.8 × 10^4^	1.4 × 10^3^	4.5	–
	P(3HB‐*co*‐3HPi)‐Cu	<2.5	<2.5	<2.5	0.4	4.1

**FIGURE 4:**
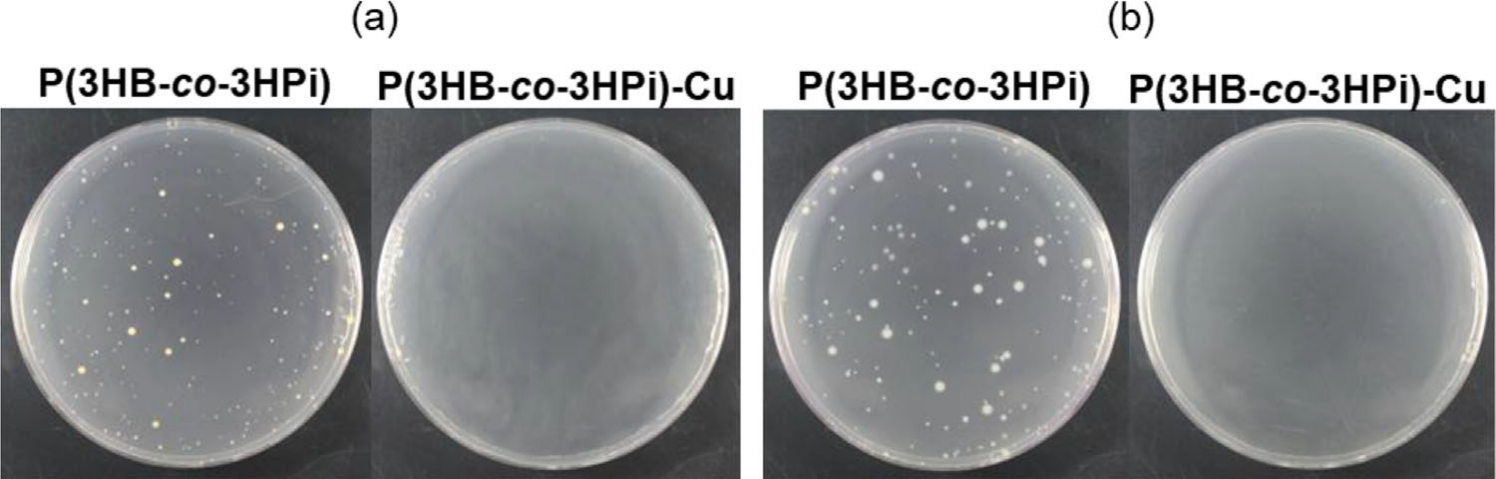
Colony formation on agar medium after antibacterial tests for P(3HB‐*co*‐3HPi) films and P(3HB‐*co*‐3HPi)‐Cu films: (a) *Staphylococcus aureus* and (b) *Escherichia coli*.

The calculated *R* values were 2.3 for *Staphylococcus aureus* and 4.1 for *Escherichia coli*, indicating that the P(3HB‐*co*‐3HPi)‐Cu films displayed antibacterial activity against both bacterial species. Figure 4 shows colony formation on an agar medium after the test. Compared with the P(3HB‐*co*‐3HPi) films, the P(3HB‐*co*‐3HPi)‐Cu films showed significantly fewer colonies for both *Staphylococcus aureus* and *Escherichia coli*, indicating that microbial growth was suppressed. This antibacterial activity could be likely due to the generation of ROS as a result of the bacterial contact with Cu^2+^ ions, although there is no direct supporting evidence [17]. Therefore, loading Cu^2+^ ions onto the surface of oxidized P(3HB‐*co*‐3HPi) films induced antimicrobial activity against both Gram‐positive and Gram‐negative bacteria.

**FIGURE 5:**
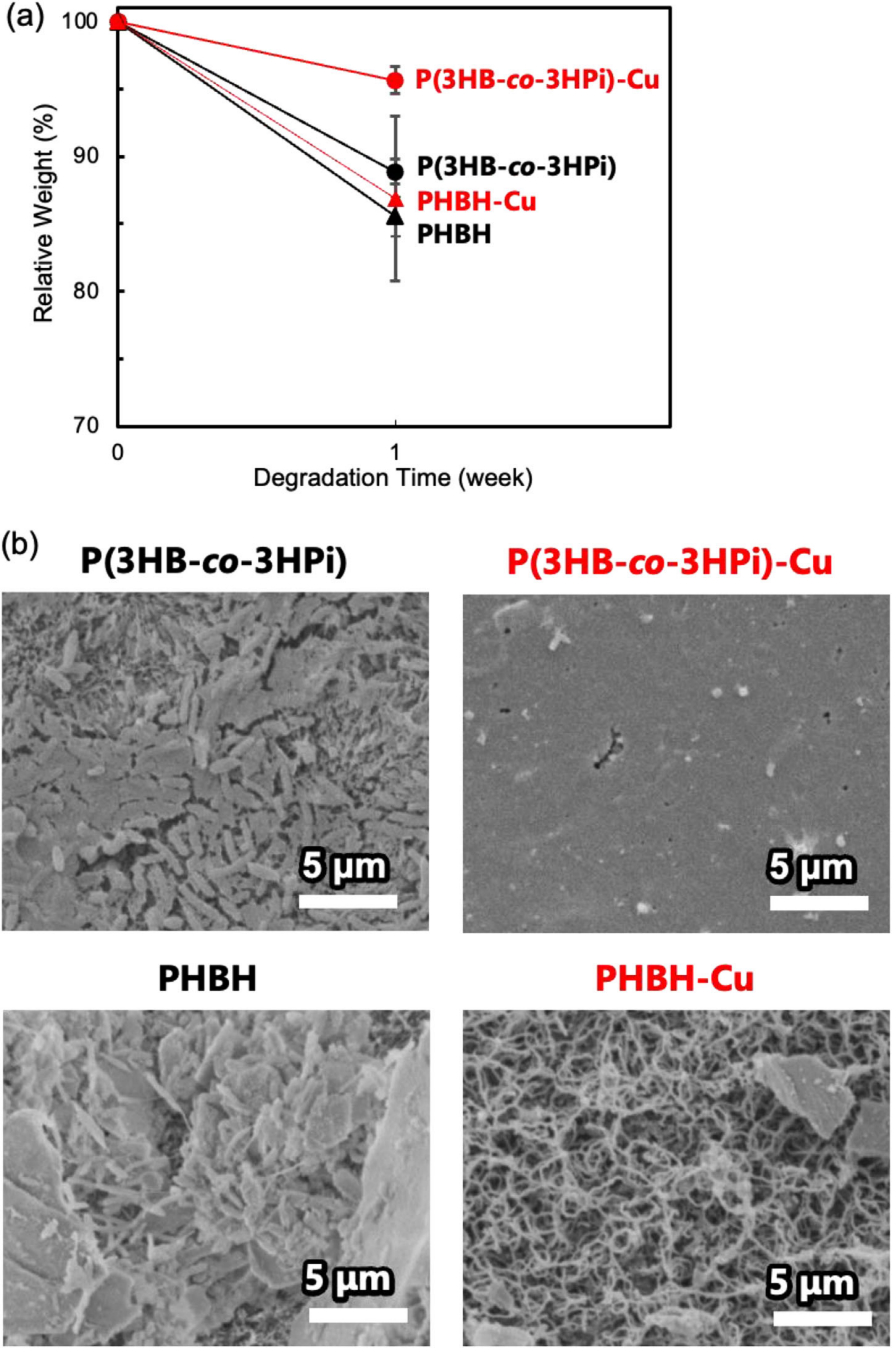
Environmental degradation tests in seawater for P(3HB‐*co*‐3HPi), P(3HB‐*co*‐3HPi)‐Cu, PHBH, and PHBH‐Cu films: (a) relative weight change in the films and (b) surface observation of the films using SEM after 1 week.

### 2.3 Biodegradation Test in Seawater

The biodegradation of P(3HB‐*co*‐3HPi), P(3HB‐*co*‐3HPi)‐Cu, PHBH, and PHBH‐Cu films was tested in seawater. After 1 week, the P(3HB‐*co*‐3HPi)‐Cu film showed significantly reduced weight loss than the P(3HB‐*co*‐3HPi) film, while the PHBH and PHBH‐Cu films showed minimal difference in weight loss (Figure 5a). The scanning electron microscopy (SEM) observations of the film surfaces after one week showed that the P(3HB‐*co*‐3HPi)‐Cu film was not covered in biofilm, and the surface remained smooth. In contrast, the PHBH‐Cu film was covered in biofilm, and the surface was degraded (Figure 5b). In the case of P(3HB‐*co*‐3HPi)‐Cu films, the antimicrobial activity of Cu^2+^ ions inhibited the growth of PHA‐degrading bacteria in the seawater, as observed in the antibacterial tests. In contrast, in the case of PHBH‐Cu films, because Cu^2+^ ions were absent, as confirmed in Figure 2b, biodegradation occurred because microbial growth was not inhibited. This result reinforced that P(3HB‐*co*‐3HPi) was more effective in loading divalent metal cations.

**FIGURE 6:**
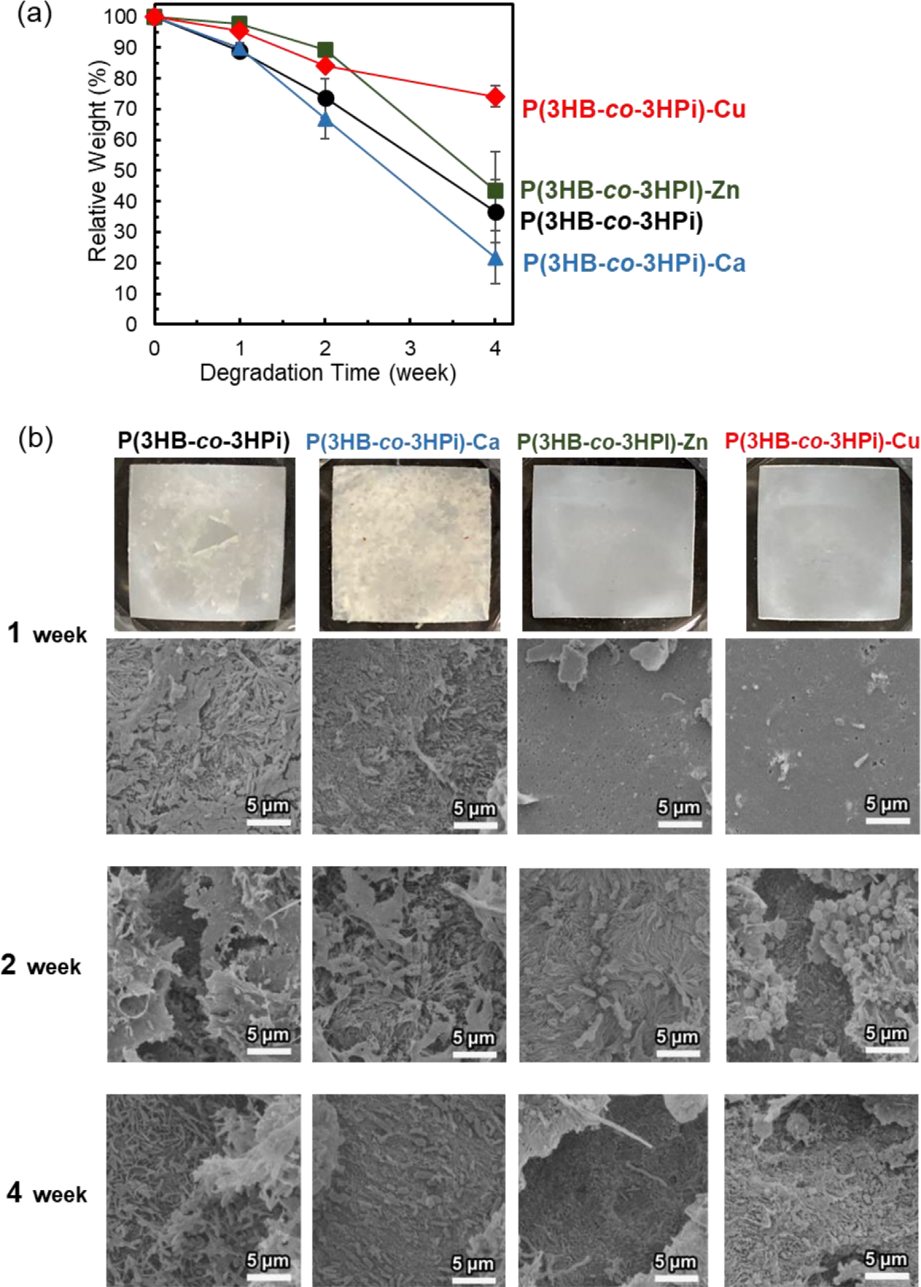
Environmental degradation tests in seawater for P(3HB‐*co*‐3HPi), P(3HB‐*co*‐3HPi)‐Ca, P(3HB‐*co*‐3HPi)‐Zn, and P(3HB‐*co*‐3HPi)‐Cu films: (a) relative weight change in the films and (b) appearance and surface observation of the films using SEM after 1, 2, and 4 weeks.

Then, longer‐term biodegradation tests were conducted in seawater on untreated P(3HB‐*co*‐3HPi) films and P(3HB‐*co*‐3HPi) films loaded with the three types of divalent cations (Cu^2+^, Zn^2+^, and Ca^2+^). The weight measurements of these films showed differences between samples. The P(3HB‐*co*‐3HPi) and P(3HB‐*co*‐3HPi)‐Ca films showed approximately 10% weight loss after 1 week and continued to lose significant weight after 2 and 4 weeks (Figure 6a). Biofilm formation on the surface of these films was observed from the appearance and by SEM observation (Figure 6b). In addition, surface observation of these films after 2 and 4 weeks showed dense biofilm formation and surface degradation.

In contrast, the P(3HB‐*co*‐3HPi)‐Cu and P(3HB‐*co*‐3HPi)‐Zn films showed weight loss of only a few percent after 1 week (Figure 6a). Based on the appearance of these films, the occurrence of biofilm formation on the films after 1 week was detected, and no biofilm formation was observed when using SEM (Figure 6b). After 2 weeks, these films showed less weight loss than the P(3HB‐*co*‐3HPi) and P(3HB‐*co*‐3HPi)‐Ca films; however, SEM observation showed biofilm formation and surface degradation of the films. After 4 weeks, the P(3HB‐*co*‐3HPi)‐Zn film experienced similar weight loss as the untreated P(3HB‐*co*‐3HPi) film, while the P(3HB‐*co*‐3HPi)‐Cu film showed approximately 25% weight loss. Potentially, the biodegradation rate was reduced for Cu^2+^ ions compared with that for Zn^2+^ ions because of the higher antimicrobial activity of Cu^2+^ ions [22].

These results indicate that the loading of Cu^2+^ and Zn^2+^ ions inhibited the biodegradation of P(3HB‐*co*‐3HPi) films in seawater for 1 week because of the antimicrobial properties of these cations. Although biodegradation of these films occurred after 2 weeks, the cations suppressed the biodegradation rate, especially for Cu^2+^ ions. Furthermore, because the films loaded with Ca^2+^ ions, which lack antibacterial activity, were degraded, the loading of divalent cations with antimicrobial properties is effective in inhibiting the biodegradation of PHA‐based films in seawater.

## 3 Conclusion

In this study, divalent metal cations were loaded onto the surface of PHA films oxidized using photoactivated ClO_2_• gas. Cu^2+^ ions were loaded onto P(3HB‐*co*‐3HPi) films, PHAs with two adjacent methyl groups on the side chain. In addition, Zn^2+^ and Ca^2+^ ions were loaded onto P(3HB‐*co*‐3HPi) films. In seawater, the biodegradation of P(3HB‐*co*‐3HPi) films loaded with Cu^2+^ and Zn^2+^ ions, which have antibacterial properties, was initially inhibited and occurred gradually with time. In other words, loading antimicrobial metal divalent cations inhibited biodegradation in seawater temporarily but allowed biodegradation to initiate with time. In conclusion, this study expands the potential application of PHAs in seawater to fishing gear, such as fishing lines that are repeatedly used in the ocean for short periods of time.

## 4 Experimental Section

### 4.1 Materials

P(3HB‐*co*‐3HPi) containing 6 mol% 3‐hydroxypivalic acid was biosynthesized using a method described in a previous study [23]. PHBH containing 9 mol% 3‐hydroxyhexanoic acid was provided by Kaneka Corporation. P(3HB‐*co*‐3HPi) and PHBH were purified by dissolution in chloroform and reprecipitation in hexane. Sodium chlorite was purchased from Sigma–Aldrich. 10 m hydrochloric acid, copper bromide, zinc bromide, and calcium chloride were purchased from Fujifilm Wako Pure Chemical.

The P(3HB‐*co*‐3HPi) and PHBH films were prepared by hot‐pressing the powders for 30 s at 1 MPa and 30 s at 5 MPa at 180°C and 150°C, respectively. The average thickness of these films was approximately 170 µm.

### 4.2 Surface Oxidation of Films

In accordance with previous studies, P(3HB‐*co*‐3HPi) and PHBH films were oxidized at room temperature using ClO_2_• gas activated by UV (λ = 365 nm, 20 mW/cm^2^) irradiation [19, 20]. ClO_2_• gas was generated by adding 0.1 mL of 10 m HCl to a 1.4 × 10^−2^ mg/mL sodium chlorite solution. The UV irradiation treatment time was 10 min, and oxidation was performed on both sides of the films. After oxidation, all samples were rinsed thoroughly with deionized water and dried at room temperature.

The surface chemical compositions of the films before and after oxidation were analyzed using an X‐ray photoelectron spectrometer (JPS‐9010, JEOL, Japan) equipped with monochromatic Mg Kα as the X‐ray source. The high‐resolution XPS spectra of C 1s were measured in 0.1 eV energy steps with a fixed pass energy of 10 eV. The binding energies of the C 1s peaks were referenced to the C─H (sp^3^) carbon set at 284.7 eV.

### 4.3 Molecular Weight Analysis

The molecular weight of P(3HB‐*co*‐3HPi) powders and films was measured by GPC using the method described by Qie et al. [24]. The samples were prepared by dissolving the polymers in chloroform with hexafluoroisopropanol (HFIP) (v/v = 1:1) at 2 mg/mL concentration and filtering them using a 0.45 µm PVDF membrane. GPC analysis was performed using a Shimadzu Nexera 40 GPC system (Shimadzu, Kyoto, Japan) and a Shodex RI‐504 refractive index detector (Showa Denko, Tokyo, Japan) equipped with two joint columns of KF‐406LHQ. 300 mM HFIP in chloroform was used as the eluent at a flow rate of 0.3 mL/min, and the oven temperature of the column was set at 40°C. Polystyrene standards were used to construct the calibration curves.

### 4.4 Loading of Divalent Metal Cations

The oxidized P(3HB‐*co*‐3HPi) and PHBH films were immersed in a 10 mg/mL copper (II) bromide aqueous solution for 2 h and washed in deionized water for 1 h. These P(3HB‐*co*‐3HPi) and PHBH films were abbreviated as P(3HB‐*co*‐3HPi)‐Cu film and PHBH‐Cu film, respectively. The surfaces of these films were analyzed for Cu atoms using XPS. For P(3HB‐*co*‐3HPi), the oxidized films were immersed in aqueous solutions of 10 mg/mL zinc (II) bromide or 10 mg/mL calcium chloride and washed with deionized water. These films are abbreviated as P(3HB‐*co*‐3HPi)‐Zn film and P(3HB‐*co*‐3HPi)‐Ca film, respectively. The surfaces of these films were also analyzed using XPS for Zn and Ca atoms.

### 4.5 Antibacterial Tests

To evaluate the antibacterial activity of the P(3HB‐*co*‐3HPi) and P(3HB‐*co*‐3HPi)‐Cu films against Gram‐positive and Gram‐negative bacteria, an antibacterial test was performed according to the film adhesion method, JIS Z 2801, 2012. In this method, the tested film is inoculated with a bacterial solution and covered with polyethylene (PE) film to disperse the solution across the film surface. After 24 h, the bacteria adhered to the films were washed off, and the number of viable bacteria was counted on an agar medium to compare the change in bacterial concentration before and after the test. The size of each sample was 2.5 cm × 2.5 cm, and the size of the covering PE film was 2.0 cm × 2.0 cm. The inoculated bacterial solution contained *Staphylococcus aureus* (NBRC12732) and *Escherichia coli* (NBRC3972) as representatives of Gram‐positive and Gram‐negative bacteria, respectively. The concentration of the bacterial solution was 7.0 × 10^5^ cfu/mL, and the inoculum volume was 0.1 mL. Prior to testing, the film surface was sterilized using irradiation with UV light at 254 nm (5 µW/cm^2^) for 15 min. The test was conducted in the dark at 35°C and relative humidity greater than 90% and was repeated in triplicate for each sample.

### 4.6 Biodegradation Tests in Seawater

The biodegradability of the P(3HB‐*co*‐3HPi), P(3HB‐*co*‐3HPi)‐Cu, PHBH, and PHBH‐Cu films was investigated in seawater. For P(3HB‐*co*‐3HPi), the biodegradability of the P(3HB‐*co*‐3HPi)‐Zn and P(3HB‐*co*‐3HPi)‐Ca films was also investigated. Seawater samples were collected into 2 L screw‐cap bottles from Odaiba Seaside Park (Tokyo, Japan). The films were placed in the bottles, and the cap of the bottle was loosely opened to allow air to pass through. The test was conducted in an incubator at 25°C for up to 4 weeks. The biodegradability of the films was evaluated by measuring the change in weight after washing with deionized water and vacuum drying at room temperature. If biofilm adhesion prevented the films from being washed with water, they were gently washed with an ultrasonic cleaner. Four replicate films (1 cm × 1 cm) were prepared for each sample, three for weighing and one for surface observation using scanning electron microscopy (JCM‐700, JEOL, Japan). Before observation, the surface was coated with gold using an ion sputtering system (MSP‐1S, VACUUM DEVICE, Japan).

## Conflicts of Interest

The authors declare no conflict of interest.

## Supporting information




**Supporting file**: mabi70069‐sup‐0001‐SuppMat.docx

## Data Availability

The data that support the findings of this study are available from the corresponding author upon reasonable request.
