# Supplementary material for: Effect of Antimicrobial Divalent Metal Cations Onto Oxidized Surface of Polyhydroxyalkanoate Films on Biodegradability in Seawater
Source: Macromol Biosci. 2025 Aug 26;25(12):e00162. doi: 10.1002/mabi.202500162 (PMC12704234; doi:10.1002/mabi.202500162)
Supplement: Supplementary file 1 — Supporting file: mabi70069‐sup‐0001‐SuppMat.docx [file MABI-25-e00162-s001.docx]

**Supporting Information**

**Effect of Antimicrobial Divalent Metal Cations onto Oxidized Surface of Polyhydroxyalkanoate Films on Biodegradability in Seawater**

*Jobu Tateiwa, Yu-I Hsu, Hiroshi Uyama, Takeharu Tsuge, Tadahisa Iwata**

J. Tateiwa, T. Iwata

Department of Biomaterials Sciences, Graduate School of Agricultural and Life Sciences, The University of Tokyo, 1-1-1 Yayoi, Bunkyo-ku, Tokyo 113-8657, Japan

*E-mail: atiwata@g.ecc.u-tokyo.ac.jp

Y. Hsu, H. Uyama

Department of Applied Chemistry, Graduate School of Engineering, Osaka University, 2-1 Yamadaoka, Suita, Osaka 565-0871, Japan

T. Tsuge

Department of Materials Science and Engineering, Institute of Science Tokyo, 4259 Nagatsuta, Midori-ku, Yokohama, Kanagawa 226-8502, Japan


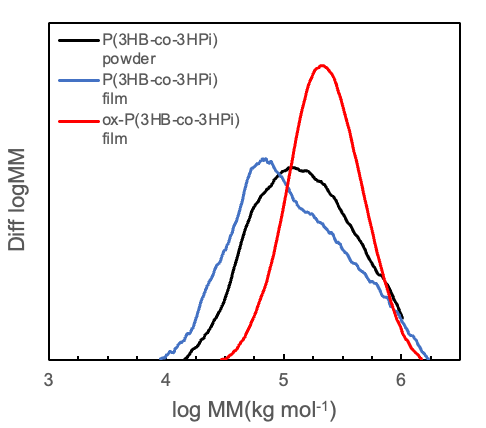


**Figure S1.** Molecular weight distribution of P(3HB-*co*-3HPi) powder and films after processing and surface oxidation.
